# Controlling for population structure and genotyping platform bias in the eMERGE multi-institutional biobank linked to electronic health records

**DOI:** 10.3389/fgene.2014.00352

**Published:** 2014-11-04

**Authors:** David R. Crosslin, Gerard Tromp, Amber Burt, Daniel S. Kim, Shefali S. Verma, Anastasia M. Lucas, Yuki Bradford, Dana C. Crawford, Sebastian M. Armasu, John A. Heit, M. Geoffrey Hayes, Helena Kuivaniemi, Marylyn D. Ritchie, Gail P. Jarvik, Mariza de Andrade, the electronic Medical Records and Genomics (eMERGE) Network

**Affiliations:** ^1^Division of Medical Genetics, Department of Medicine, University of WashingtonSeattle, WA, USA; ^2^Department of Genome Sciences, University of WashingtonSeattle, WA, USA; ^3^The Sigfried and Janet Weis Center for Research, Geisinger Health SystemDanville, PA, USA; ^4^Department of Biochemistry and Molecular Biology, Center for Systems Genomics, Pennsylvania State UniversityUniversity Park, PA, USA; ^5^Center for Human Genetics Research, School of Medicine, Vanderbilt UniversityNashville, TN, USA; ^6^Department of Molecular Physiology and Biophysics, Vanderbilt UniversityNashville, TN, USA; ^7^Division of Biomedical Statistics and Informatics, Mayo ClinicRochester, MN, USA; ^8^Division of Cardiovascular Diseases, Mayo ClinicRochester, MN, USA; ^9^Division of Endocrinology, Metabolism, and Molecular Medicine, Feinberg School of Medicine, Northwestern UniversityChicago, IL, USA

**Keywords:** principal component analysis, ancestry, biobank, loadings, genetic association study

## Abstract

Combining samples across multiple cohorts in large-scale scientific research programs is often required to achieve the necessary power for genome-wide association studies. Controlling for genomic ancestry through principal component analysis (PCA) to address the effect of population stratification is a common practice. In addition to local genomic variation, such as copy number variation and inversions, other factors directly related to combining multiple studies, such as platform and site recruitment bias, can drive the correlation patterns in PCA. In this report, we describe the combination and analysis of multi-ethnic cohort with biobanks linked to electronic health records for large-scale genomic association discovery analyses. First, we outline the observed site and platform bias, in addition to ancestry differences. Second, we outline a general protocol for selecting variants for input into the subject variance-covariance matrix, the conventional PCA approach. Finally, we introduce an alternative approach to PCA by deriving components from subject loadings calculated from a reference sample. This alternative approach of generating principal components controlled for site and platform bias, in addition to ancestry differences, has the advantage of fewer covariates and degrees of freedom.

## 1. Introduction

To reach the statistical power needed for genome-wide association studies, large numbers of participants are needed. This can be achieved through large research networks such as the Electronic Medical Records and Genomics (eMERGE) Network, which comprises a multi-ethnic cohort of ~57,000 participants linked to electronic health records (EHRs) for phenotype mining from nine participating sites (seven adult; two pediatric) in the United States (U.S.) (Gottesman et al., 2013). When combining genetic data from diverse data sets, understanding the contribution of ancestry, genotyping platform, and site bias are of vital importance.

Through the course of the eMERGE project, multiple genotyping platforms from both Illumina and Affymetrix were utilized (Gottesman et al., 2013; Crawford et al., 2014). Imputation using the BEAGLE software was then carried out to allow merging of the diverse data sets (Verma et al., *Imputation and quality control steps for combining multiple genome-wide data sets*. Manuscript submitted for publication).

There were ancestry or racial/ethnic differences both within and across the eMERGE Network sites in addition to the platform heterogeneity. The majority of eMERGE study sites based race/ethnicity on self-report while Vanderbilt University's BioVU used third-party or administratively assigned race/ethnicity (Dumitrescu et al., 2010). The major group for the entire eMERGE sample set is of European-descent. eMERGE also includes a sizeable African-descent and Hispanic sample (Gottesman et al., 2013). The latter represents a three-way admixture event (Manichaikul et al., 2012) that further contributes to expected ancestral differences within and across eMERGE. There are also both cryptic and known related participants, especially in Marshfield Clinic Research Foundation (Gottesman et al., 2013; Crawford et al., 2014).

We present an example of integrating the diverse genetic data sets from the eMERGE Network in a systematic fashion and provide guidance for other investigators in large research networks. We outline a general approach for selecting variants for input into a sample variance-covariance matrix on the adult participants in eMERGE, the conventional principal component analysis (PCA) approach in human genetics research (Patterson et al., 2006). We also describe how we categorized genetic ancestry based on self-reported race, framed in terms of continental origin, in line with standard protocol in human genetic research (NHGRI, 2005; Ali-Khan et al., 2011).

Given our “sizeable” non-European sample in the presence of platform bias and imputation, the eMERGE Network took great care in not only assessing and adjusting for ancestry, but also exploring alternative methods to do so and increase power. To assess ancestry in related individuals, Zhu et al. (2008) introduced a method of generating principal components (PCs) by deriving SNP loadings from founders, and applying them to the entire sample. We introduce this concept of deriving SNP loadings from the BEAGLE imputation 1000 Genomes reference sample, and apply it to the entire imputed sample set of 57,000 genotyped individuals from the eMERGE Network as an alternative approach to control for site and platform bias in addition to ancestry differences for our large cohort.

## 2. Materials and methods

The eMERGE Network comprises a multi-ethnic cohort of ~57,000 participants linked to EHRs for phenotype mining from nine participating sites (seven adult; two pediatric) in the United States (Gottesman et al., 2013) with genotype and imputed data.

### 2.1. Imputation

The imputation and merging were performed by the eMERGE Coordinating Center (CC) at Pennsylvania State University (PSU). Detailed quality assurance/quality control (QA/QC) measures are outlined in the imputation guide provided on the PSU eMERGE CC web site (see Web Resources). Before imputation, study site data were converted to the same build (Build 37) as the imputation reference data set. Next, strand flipping was employed to account for different strand alignments including Illumina TOP/BOT strand, plus(+) / minus(−), and forward/reverse (Nelson et al., 2012). Finally, phasing and imputation were performed on randomized ancestry sub-samples against a “Cosmopolitan” reference set from the 1000 Genomes containing multiple ancestry groups provided by the BEAGLE software package (Browning and Browning, 2009). While the imputation data presented are derived from using BEAGLE software (Browning and Browning, 2009), it should be noted that IMPUTE2 software (Howie et al., 2012) produced nearly identical results (see Supplementary Figure S1) (Howie et al., 2011; Delaneau et al., 2013).

### 2.2. PCA

There are multiple software packages for running PCA to estimate genomic ancestry, but we utilized the high-performance computing toolset SNPRelate R package (Zheng et al., 2012) for multiple reasons. First, the increased computational performance allows for PCA analyses of a large number of participants such as eMERGE. Second, this tool allows the extraction of both sample and SNP loadings, which allows the correction of population stratification for related and unrelated participants (Zhu et al., 2008). The two types of matrices are mathematically equivalent and can be derived from one another. Finally, SNPRelate allows for absolute genotype-PC correlation to assess whether a local region of the genome is driving the correlation structure (Zheng et al., 2012).

We derived PCs using three general approaches, each applied to the overall set and to each ancestry group. First, we performed PCA on a combined data set (across sites) after imputation using the BEAGLE software package (Version 3.3.1) (Browning and Browning, 2009). Second, we performed PCA on a pre-imputed merged version (across sites) of the data. Finally, we derived PCs for the entire set using SNP loadings generated from the BEAGLE imputation reference set (Browning and Browning, 2009).

For all genotype data used to generate the variance-covariance matrices and to eliminate redundant SNPs in high linkage disequilibrium (LD), we applied the following thresholds. The autosomal variants were selected after LD pruning at *r* > 0.5 with a 500 kbp (kilo basepairs) sliding window, and a minor allele frequency (MAF) > 0.05. In addition, a variant missingness filter of 0.02 was applied. For both PCA on the combined imputed and the combined preimputed, which is basically the singular value decomposition on the sample covariance matrix as outlined in Patterson et al. (2006).

#### 2.2.1. Deriving PCA using reference sample loadings

We also assessed PCA using the Zhu et al. (2008) method by deriving SNP loadings from the BEAGLE imputation 1000 Genomes reference sample, and applying it to the entire sample set. As such, we utilized their nomenclature with respect to generating the components. This was implemented using the SNPRelate R package (Zheng et al., 2012), specifically the snpgdsPCASampLoading and snpgdsPCASNPLoading functions (see Web Resources).

We treated the entire eMERGE cohort as one “related” family, and the imputation reference sample as (*a* = 1,2,…, *B*) unrelated. Because of this, the *g_ij_* marker genotype value of the *j*th individual in the *i*th family as utilized by Zhu et al. (2008), simplified to *g_j_*. The column vector *X_ij_* = (*x*_*j*__1_, *x*_*j*2_,…, *x*_*jM*_)^*T*^ of *l* = 1,2,…, *M* biallelic markers, and was coded as an additive model of inheritance.

The variance-covariance matrix for the marker data from the reference sample (unrelated), took on the form $\Sigma =\Sigma_{a\;=\;1}^{B}(X_{a}-\bar{X}){\left(X_{a}-\bar{X}\right)}^{T}$, assuming *X* as the overall genotype mean for those samples. Following Zhu et al. (2008), we let *e*_*l*_ be the *l*th eigenvalue of Σ, where *l* = 1,2,…, *M*, which is a vector of the SNP loadings. We then derived the *l*th PC for the individual (*j*) of the entire cohort or “related” family by *t*_*jl*_ = (*X*_*j*_ − *X*)^*T*^*e_l_*.

### 2.3. Venous thromboembolism association

The venous thromboembolism (VTE) phenotype was extracted using an EHR-driven algorithm from African ancestry participants (Pathak et al., personal communication), excluding patients with cancer. A total of 400 VTE cases and 5,065 controls were selected from 4 sites and 4 different genotype platforms (Illumina 660, 1M, and Omni; and Affymetrix 6.0). We performed two logistic regressions for association using the software PLINK v1.07 (Purcell et al., 2007). The first was adjusted for age, sex, stroke, sickle cell genetic variant, site-platform, and conventional PC1 and PC2 and the second was adjusted for age, sex, stroke, sickle cell genetic variant and “loadings” PC1 and PC2.

## 3. Results

### 3.1. Demographics

Table 1 outlines the breakdown of the 38,288 adult participants included in these analyses by eMERGE site, self-reported or administratively assigned ancestry, sex, and genotyping platform. Most sites were predominantly of European ancestry. Compared with most other eMERGE study sites, both Vanderbilt University and Northwestern University had a greater representation of African ancestry (26 and 12%, respectively). Mount Sinai School of Medicine had the greatest proportion of African ancestry (70%), followed by a sizeable proportion of Hispanic participants (19%). Overall, there were more females than males (57% vs. 43%). All sites followed this pattern, except for Geisinger Health System (53% male). Most of the genotyping across all sites was performed using Illumina arrays (610, 660, 1M and Omni), with the exception of Mount Sinai School of Medicine (Affymetrix 6.0).

**Table 1:** **Summary of eMERGE sample by self-reported ancestry, sex, and genotyping platform for the adult participants**.

	**Geisinger (*N* = 3, 111) (%)**	**Group Health (*N* = 3, 520) (%)**	**Marshfield (*N* = 4, 193) (%)**	**Mayo (*N* = 6, 836) (%)**	**Mt. Sinai (*N* = 6, 290) (%)**	**Northwestern (*N* = 4, 858) (%)**	**Vanderbilt (*N* = 9, 480) (%)**	**Combined (*N* = 38, 288) (%)**
**SELF-REPORTED ANCESTRY**								
African	0	4	0	0	70	12	26^†^	20% (7, 651)
European	99	92	99	99	11	88	66^†^	74% (28, 469)
Hispanic	0	0	0	0	19	0	0	3% (1, 258)
Other	0	5	1	0	0	0	7^†^	2% (910)
**SEX**								
Female	47	57	58	45	59	83	53	57% (21, 802)
Male	53	43	41	55	41	17	47	43% (16, 486)
**GENOTYPING PLATFORM**								
Affymetrix 6	0	0	0	0	44	0	0	7% (2, 775)
Illumina 1M	0	0	0	0	0	12	21	7% (2, 634)
Illumina 610	0	0	0	45	0	0	0	8% (3, 081)
Illumina 660	0	89	100	55	0	27	42	43% (16, 362)
Illumina Omni	100	11	0	0	56	61	37	35% (13, 436)

† *Race/ethnicity is administratively assigned*.

**Figure 1:**
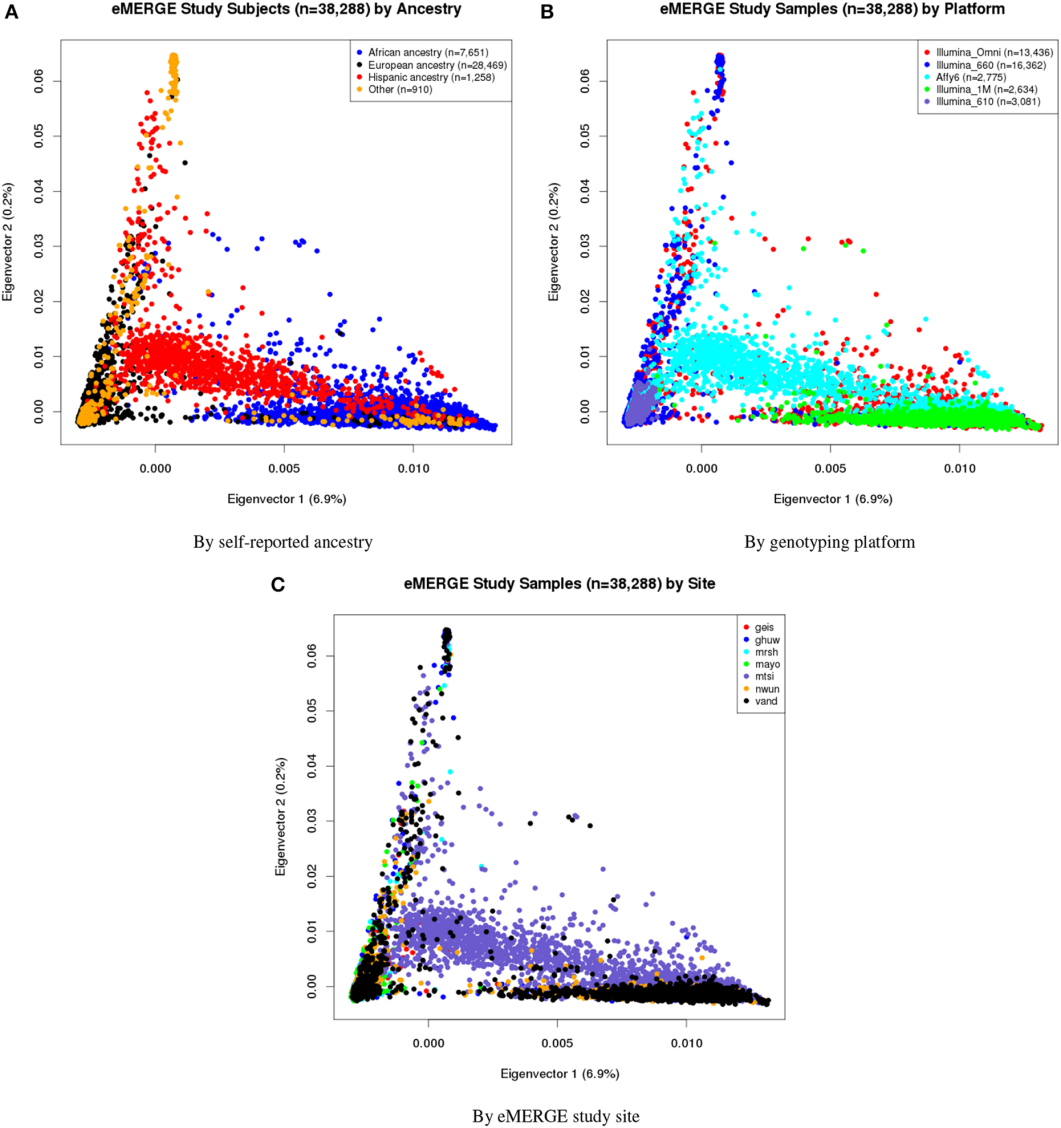
**PC plots of PCs 1 and 2 for all adults of eMERGE by self-reported race. (A), genotyping platform **(B)**, and eMERGE study site **(C)**, using BEAGLE imputed data**. (1) geis, Geisinger Health System, (2) ghuw, Group Health Research Institute/University of Washington; (3) mrsh, Marshfield Clinic Research Foundation; (4) mayo, Mayo Clinic; (5) mtsi, Mount Sinai School of Medicine; (6) nwun, Northwestern University; and (7) vand, Vanderbilt University.

**Figure 2:**
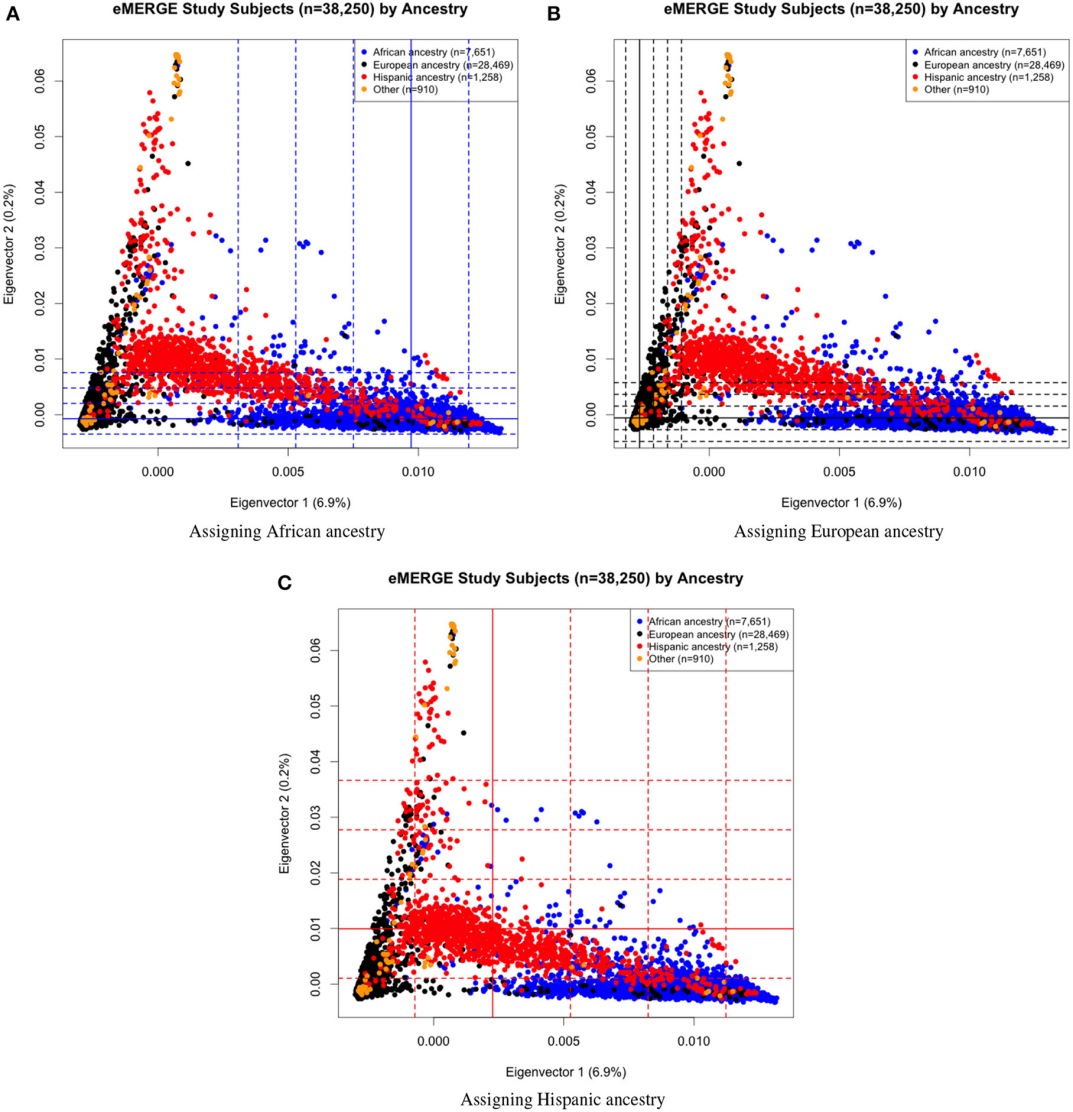
**PC plots of PCs 1 and 2 comparing eMERGE genetically determined and self-reported ancestry, using BEAGLE imputed data. (A)** African ancestry assigned using (*Q*_2_*A*__ ± 2*SD*) of eigenvectors 1 and 2 for self-reported as African ancestry. **(B)** European ancestry assigned using (*Q*_2_*E*__ ± 4*SD*) of eigenvectors 1 and 2 for self-reported as European ancestry. **(C)** Hispanic assigned using (*Q*_2_*H*__ ± 1*SD*) of eigenvectors 1 and 2 for self-reported as Hispanics.

Eigenvectors 1 and 2 for the 38,288 adult eMERGE participants are illustrated in Figure 1, annotated by self-reported race (Figure 1A), genotyping platform (Figure 1B), and by eMERGE study site (Figure 1C). Genetically determined ancestry was assigned by creating subjective boundaries for the African, European and Hispanic groups. These boundaries were set using the respective medians (*Q*_2_) and standard deviations (*SD*) calculated for each genetic ancestry group, as illustrated in Figures 2A–C for the African (*Q*_2_*A*__ ± 2*SD*), European (*Q*_2__*E*_ ± 4*SD*) and Hispanic (*Q*_2_*H*__ ± 1*SD*) groups, respectively.

### 3.2. Examination of the variance explained per PC using scree plots

**Figure 3:**
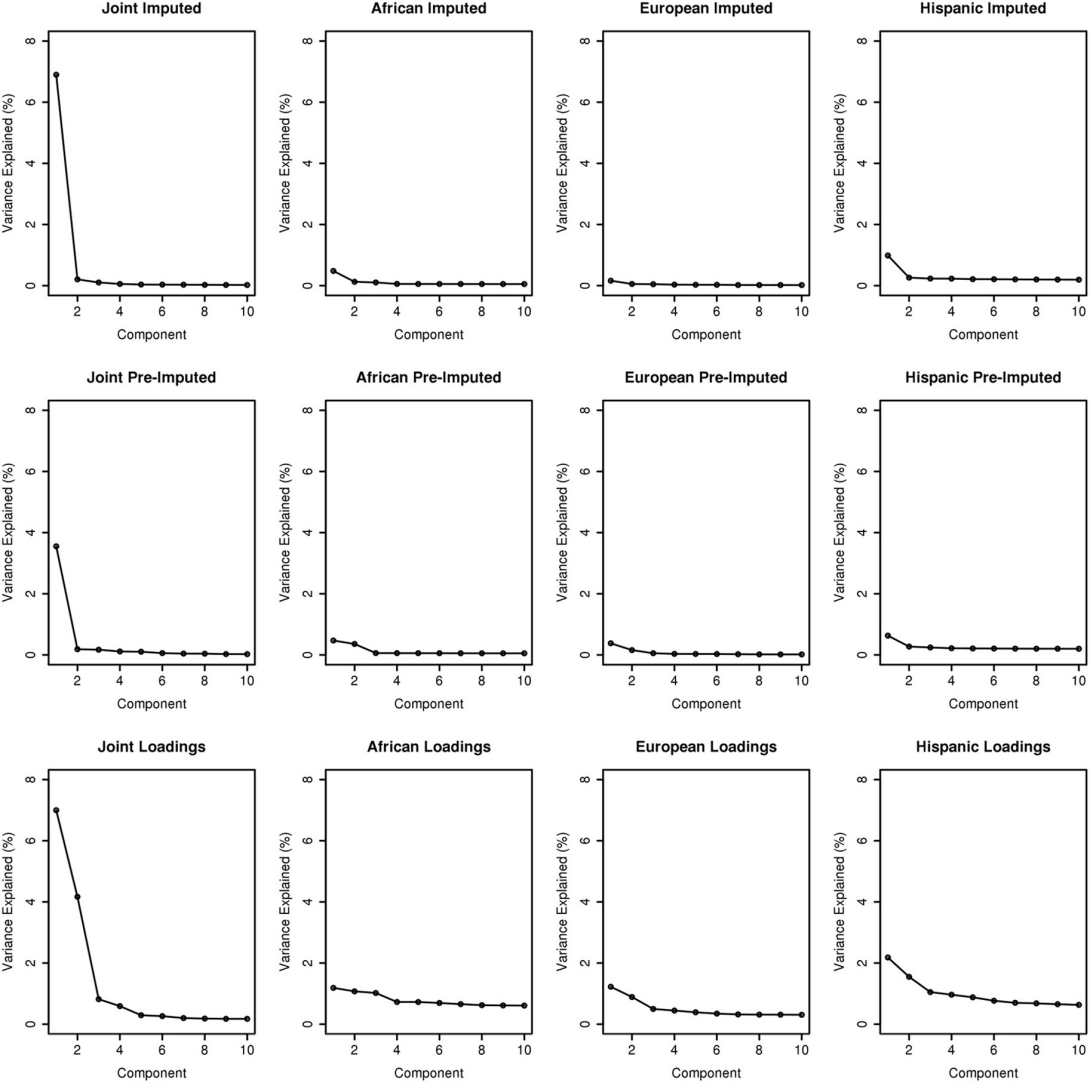
**Scree plots illustrating variance explained for PCA outlined in this manuscript**.

To assess the variance explained from each PCA, we plotted the first ten PCs against the variance explained as illustrated in Figure 3. Across the columns of the trellis we show scree plots of joint, African ancestry, European ancestry, and Hispanic groups. Across each row, we have scree plots representing PC analyses on the imputed merged set, pre-imputed merged set, and on the PC analyses using the “loadings” method outlined in Subsection 2.2.1. As expected, eigenvector 1 explains most of the variance for the joint ancestry imputed (~7%), pre-imputed (~4%), and “loadings” (~7%). When we stratified by ancestry (across the trellis), the variance explained by eigenvector 1 for the imputed and pre-imputed data sets was <1%. For the “loadings” approach with the African and European genetic ancestry data sets, the variance explained <1%, and >2% for the Hispanic group. In all scenarios (joint and all ancestry groups) the variance explained approached 0 for eigenvectors 2 through 10 for the imputed and pre-imputed data sets. Interestingly, the “loadings” approach allows for more variance explained for eigenvectors 2 and beyond, especially for the Hispanics. For the joint loadings approach, the variance explained by eigenvector 2 approached ~4%, while the genetic ancestry groups approached 1%.

### 3.3. Evaluation of the effect of ancestry on PC plots—joint and stratified ancestry

**Figure 4:**
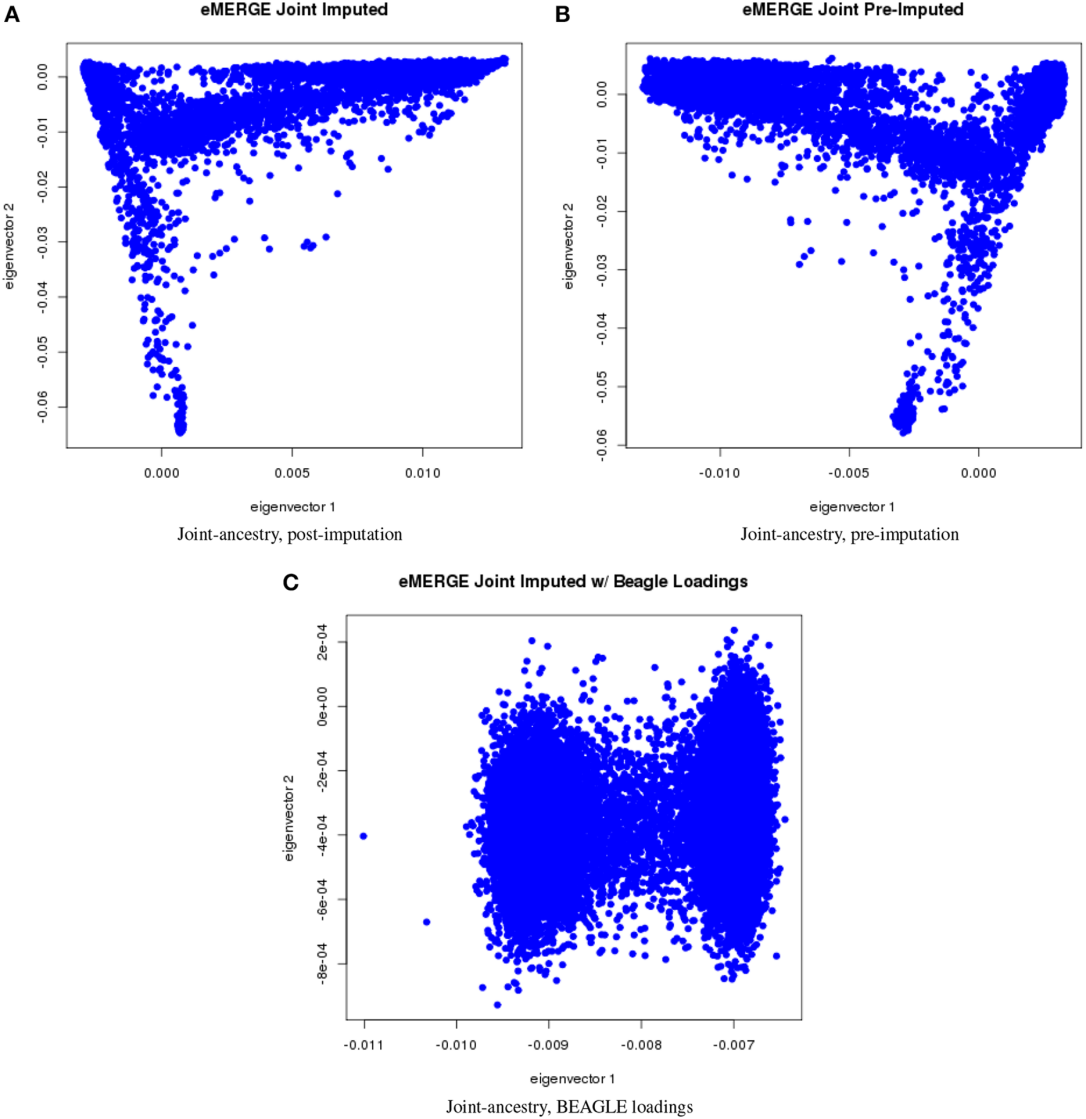
**PC plots of eMERGE joint ancestry. (A)** Plot of eigenvectors 1 and 2 for the joint imputed data set. **(B)** Plot of eigenvectors 1 and 2 for the joint pre-imputed data set. **(C)** Plot of eigenvectors 1 and 2 for the joint imputed data set using the “loadings” method.

**Figure 5:**
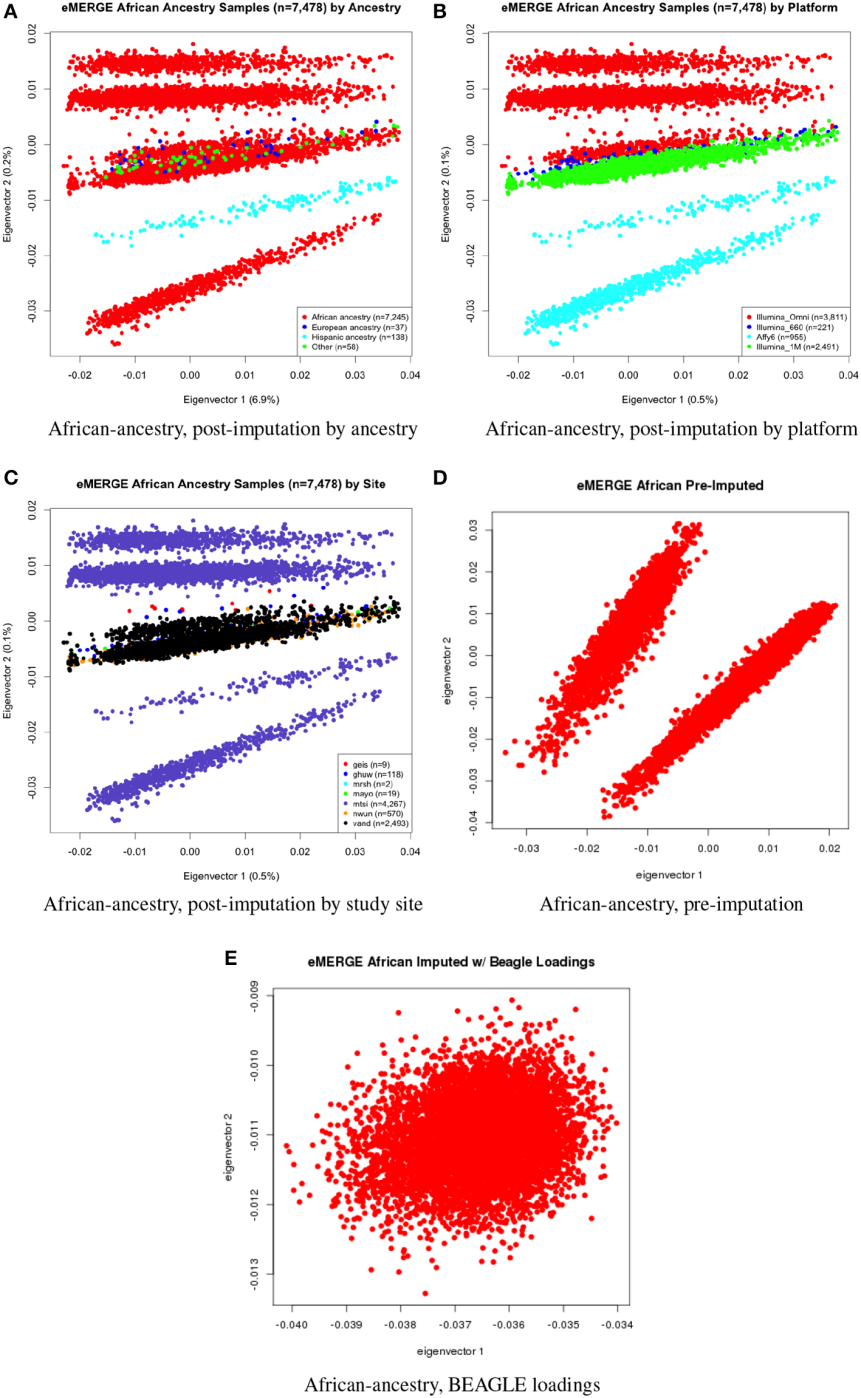
**PC plots of eMERGE participants geneticaly determined to be of African ancestry. (A)** Plot of eigenvectors 1 and 2 for the imputed data set African ancestry participants, annotated by self-reported ancestry. **(B)** Plot of eigenvectors 1 and 2 for the imputed data set African ancestry participants, annotated by genotyping platform. **(C)** Plot of eigenvectors 1 and 2 for the imputed data set African ancestry participants, annotated by eMERGE site. **(D)** Plot of eigenvectors 1 and 2 for the pre-imputed data set African ancestry participants. **(E)** Plot of eigenvectors 1 and 2 for the imputed data set African ancestry participants using the “loadings” method.

**Figure 6:**
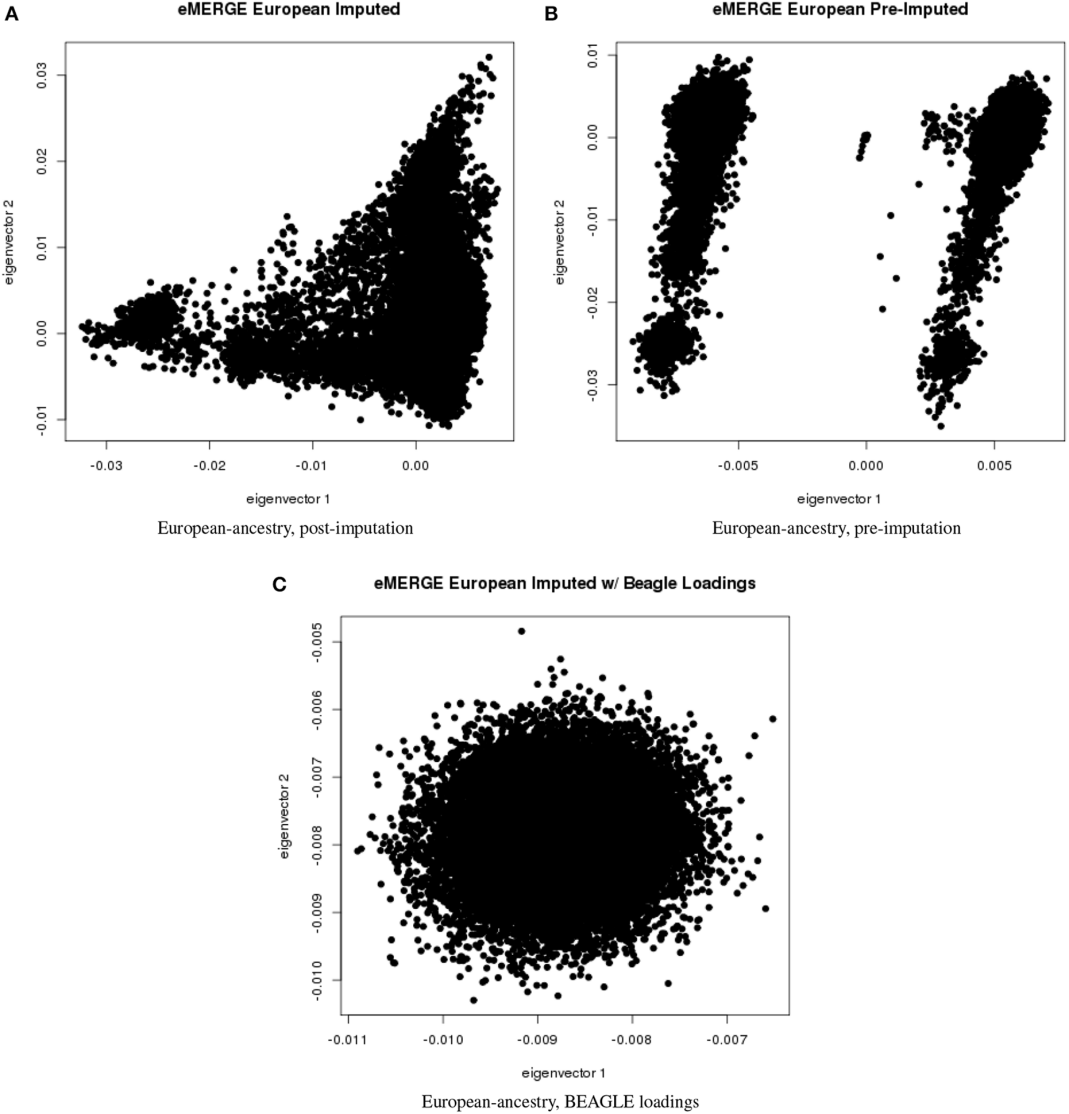
**PC plots of eMERGE participants genetically determined to be of European ancestry. (A)** Plot of eigenvectors 1 and 2 for the imputed data set Hispanic participants. **(B)** Plot of eigenvectors 1 and 2 for the pre-imputed data set Hispanic participants. **(C)** Plot of eigenvectors 1 and 2 for the imputed data set Hispanic participants using the “loadings” method.

**Figure 7:**
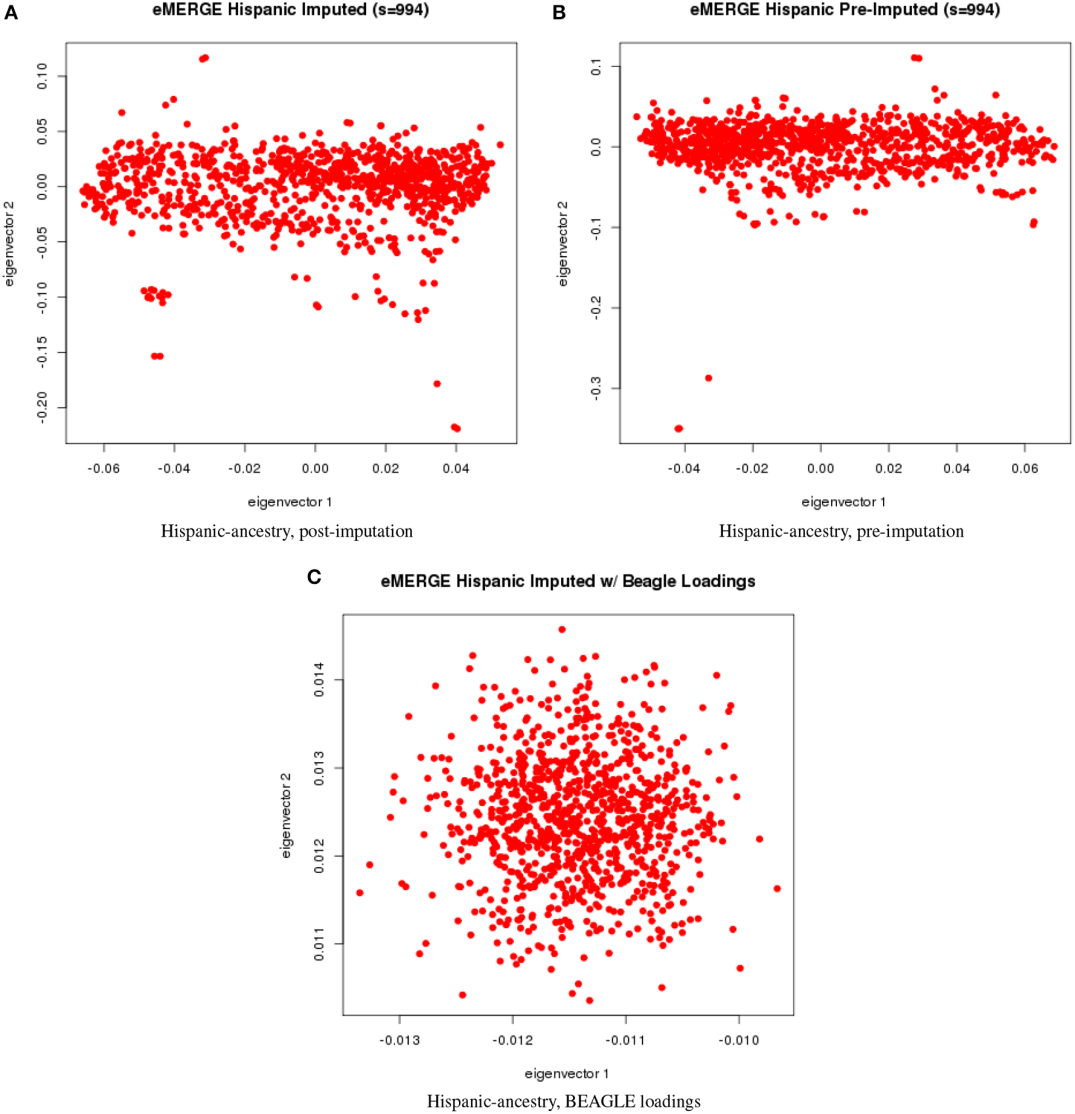
**PC plots of eMERGE participants genetically determined to be Hispanic**. **(A)** Plot of eigenvectors 1 and 2 for the imputed data set Hispanic participants. **(B)** Plot of eigenvectors 1 and 2 for the pre-imputed data set Hispanic participants. **(C)** Plot of eigenvectors 1 and 2 for the imputed data set Hispanic participants using the “loadings” method.

We evaluated the population structure by plotting eigenvectors 1 and 2 for the joint data set (Figure 4) as well as for the African (Figure 5), European (Figure 6) and Hispanic (Figure 7) ancestry groups, separately. In each case of ancestry analysis, we plotted the imputed and pre-imputed merged data set, and the data set derived from the “loadings” method. Figures 4A,B illustrate the imputation and pre-imputation data sets, respectively, and are generally opposites with respect to eigenvector 1 due to different projections for that component. Figure 4C illustrates the “loadings” data set, which offers a different characterization of the joint data set, with the African and European genetic ancestry groups largely represented by two ellipses.

Figures 5A–C illustrate the African ancestry imputation data set, annotated by self-reported race, genotyping platform, and site, respectively. As illustrated in Figures 5B,C, there are batch effects by platform and study site. The pre-imputed data set (Figure 5D) has two distinct bands for both eigenvectors 1 and 2. The “loadings” approach (Figure 5E) produces an ellipse, indicating no effect due to platform or study site. Figures 6A–C illustrate the European ancestry imputed and pre-imputed data set, and the “loadings” data set, respectively. Eigenvectors 1 and 2 for the imputed data set (Figure 6A) produce separation much like the joint ancestry analyses, while the pre-imputed data set produces two separate bands (Figure 6B). Like the African genetic ancestry “loadings” set, the European set produces an ellipse. Finally, the Hispanic data sets are illustrated in Figures 7A–C. With only 994 participants, most of the variance seems to be explained by eigenvector 1 for both the imputed (Figure 7A) and pre-imputed (Figure 7B). The “loadings” approach (Figure 7C) produces the familiar ellipse, with the mixed ancestry in the middle, most likely representing the Hispanic sample.

### 3.4. Examination of SNP-PC correlation

**Figure 8:**
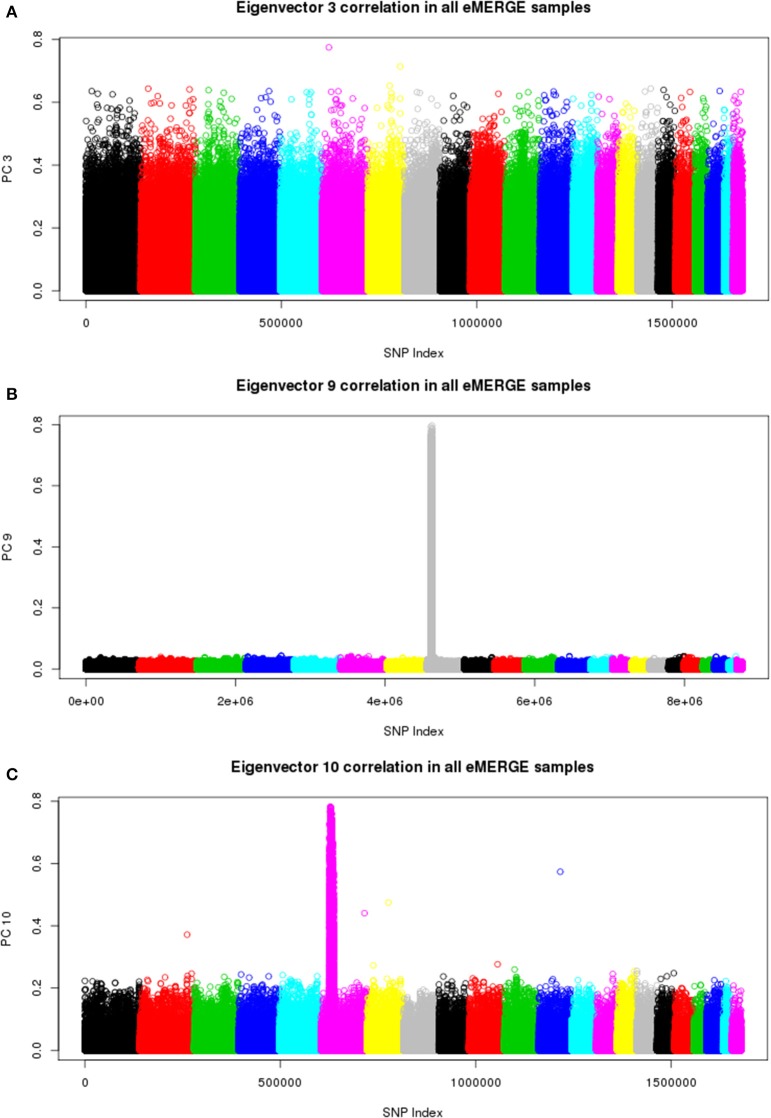
**Eigenvector-genotype correlation plots from the joint ancestry PCA analyses representing genome-wide correlation (A), correlation driven the chromosome 8 inversion (B), and correlation driven by the HLA region (C)**.

We also illustrate component-genotype absolute correlation plots generated using the SNPRelate R package for the imputed and pre-imputed data sets. Ideally, a component will be driven by genome-wide correlation patterns, as illustrated by eigenvector 3 of the pre-imputed data in Figure 8A. However, many times chromosomal artifacts will drive local regions of correlation, resulting in components dominated by that pattern. Examples of this include Figures 8B,C. Figure 8B illustrates a known chromosome 8 inversion (Feuk et al., 2006) driving the correlation patterns for eigenvector 9 in the imputed data. Figure 8C illustrates the correlation pattern driven by the HLA region for eigenvector 10 of the pre-imputed data.

### 3.5. Venous thromboembolism association

**Figure 9:**
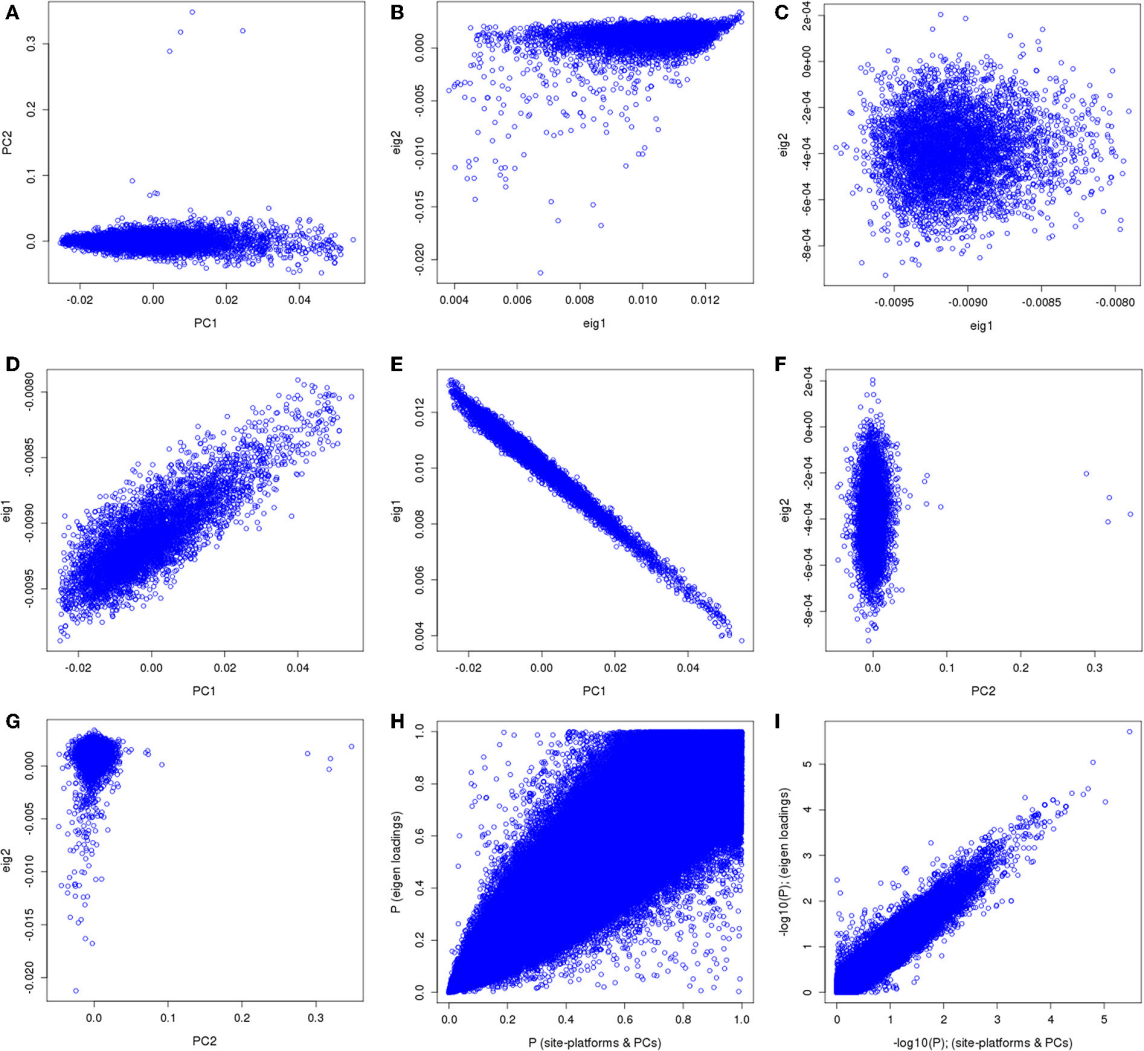
**PC comparisons derived from the “loadings” method and PCs derived from the equivalent of the imputation method for venous thromboembolism association in African ancestry participants**.

We applied our approach using the eMERGE VTE African ancestry cohort that consists of four adult sites and four genotyping platforms that had previously been analyzed controlling for site, platform and genomic ancestry (Heit et al., 2013). For clarity, the original analysis' first two eigenvectors along with site and platform will be referred to as “PCs.” The principal components derived from the imputed data set by the conventional approach will be referred as normal eigenvectors (normal “EIGs”), and derived by the “loadings” approach as “loading” eigenvectors (“loadings EIGs”). We first compared the two first PCs obtained using the eMERGE African ancestry from the original analysis with the two first eigenvectors (PCs) using the “loadings” method (Figure 9). We observed that the PCs used in the analysis had similar pattern as the standard eigenvectors (Figures 9A,B, first row), but just in a different direction for the projections. Figure 9C illustrates a bivariate normal distribution with low variance of the African genetic ancestry when using the “loadings” eigenvectors.

We observed dispersion between the first PC and the first “loading” eigenvector (Figure 9D), demonstrating that the “loadings” approach captured a different aspect of variance. The first PC showed an inverse correlation with the first PC and first normal eigenvector (Figure 9E). Such an inversion is a consequence of the arbitrary nature of mathematical sign in the computation of PCs resulting in opposite projections. Figure 9F illustrates the second PC compared to the second “loadings” PC, which shows no correlation and some outliers in the PC projection.

**Figure 10:**
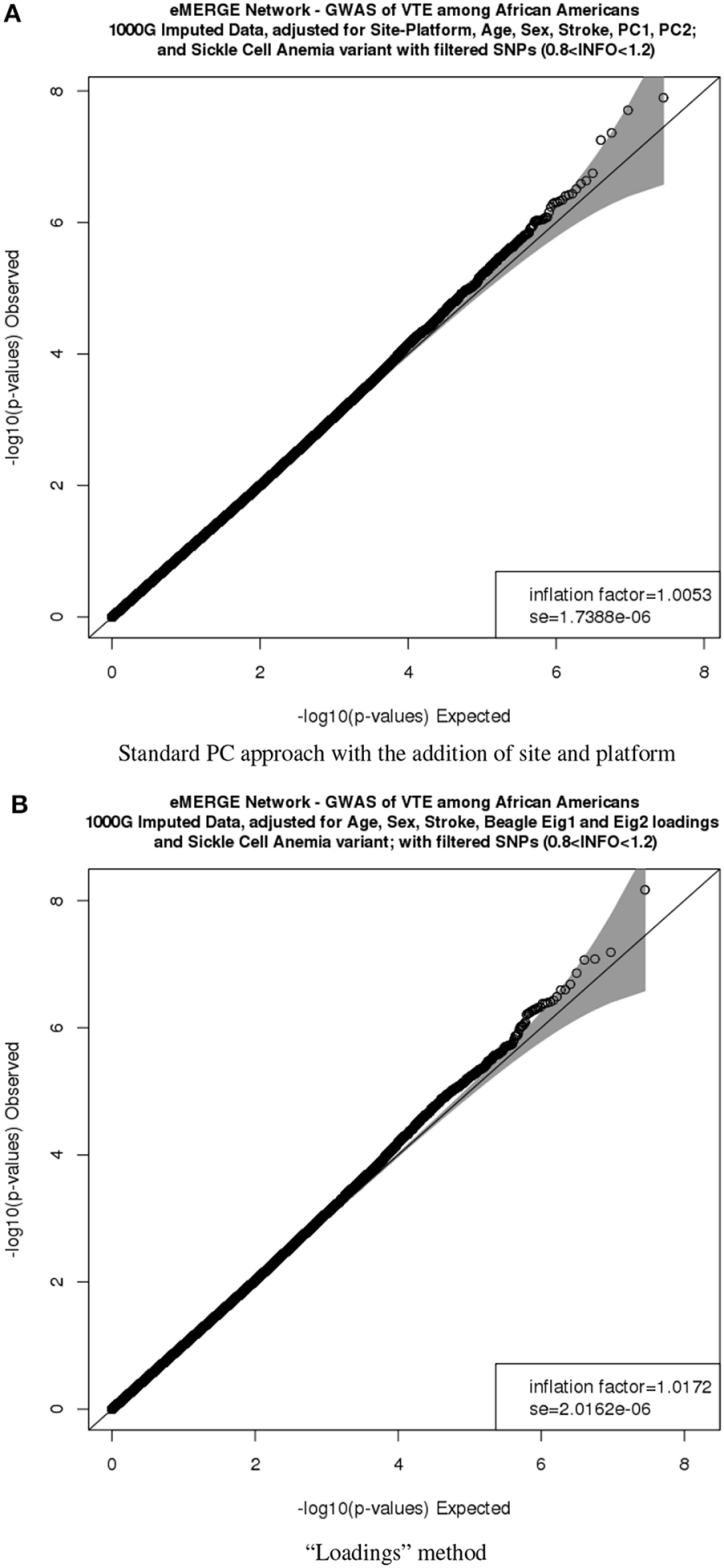
**QQ plots of the venous thromboembolism (VTE) association in African ancestry participants**. PC comparisons derived from the “loadings” method and PCs derived from the equivalent of the imputation method. **(A)** QQ plots of the VTE association in African ancestry participants using PCs derived from the equivalent of the imputation method. **(B)** QQ plots of the VTE association in African ancestry participants using PCs derived from the equivalent of the “loadings” method.

Figure 9G depicts the comparison between the second PC with the second normal eigenvector that showed the same outliers observed previously but in a different scale. Thus, by using the BEAGLE loadings we have a more parsimonious model, and the association results in *P*-values and −*log*_10_(*P*) are tighter for chromosome 22 (Figures 9H,I). Finally, Figures 10A,B represent the QQ plots for the conventional PC adjusting for site and platform method (λ = 1.01) and the “loadings” approach (λ = 1.02), respectively.

## 4. Discussion

Imputation depends on how well the genotype data (the observed LD) capture the true underlying LD. The more completely LD is represented, the more accurately the imputation will extend the LD to non-genotyped markers. There is always an inherent risk that the imputed genotypes will not represent the true state of nature accurately; this risk increases as the genotyped density decreases and the genotypes do not capture the underlying LD. We detected effects from the genotyping platform when performing the PCA (here we use platform to indicate the design as well as the method). The effect was most evident when a low-density platform such as the MetaboChip (data not shown) were combined with high-density platforms: the MetaboChip data set was an outlier even at overview scale. Platform differences re-appear when PCA is performed on apparently homogeneous subsets, e.g., African and European genetic ancestry subsets. These platform differences in homogeneous racial groups are amplified as the overall variance in the data set diminishes. Some of the differences might actually reflect subtle differences in LD in the populations due to ethnic stratification correlated with platform, because the populations were not randomly represented in the Biobank and therefore not randomized to platform.

In addition to difference of LD capture by platform, genotype encoding remains problematic when combining large data sets genotyped at different sites and on different platforms. A number of tools, e.g., liftOver (Hinrichs et al., 2006), can be used to standardize the allele states between data sets. Nevertheless, coding remains fraught with problems (Nelson et al., 2012). One data set was initially submitted with non-standard coding resulting in the data set being an outlier even with respect to other data sets on the same platform and chip. Such miscoding results in an extreme form of platform bias, as the LD is misrepresented. Other potential source of bias could be induced by the sites or genotyping center.

It is likely that the imputed data can exaggerate some underlying features. Any chromosomal variation that is poorly represented in the reference set can lead to more uniformity around the variation that causes that chromosome to be selected. Some regions that are promoted (occur prominently in a lower number PC), probably are reflecting rare chromosomes in the reference panel.

We have outlined a general checklist for filtering variants to be utilized with PCA: (1) Ensure uniformity of strand representation among different platforms to avoid the bias induced by site; (2) Select variants on autosomal chromosomes only, no sex chromosomes; (3) Filter variants with LD pruning (*r* = 0.50 − 0.84), in a sliding window of 500 kbp; (4) Filter variants on MAF >0.05, and for missingness <0.02; and (5) Examine plots of absolute correlation between PC and genotype as illustrated in Figure 10 and remove regions where chromosome artifacts (e.g., HLA, chromosome 8 inversion) are driving the correlation pattern for a given component (Laurie et al., 2010). However, in many cases removing the HLA region will not completely eliminate the correlation pattern in that region (data not shown). Normally the first ten eigenvectors are appropriate, but this depends on the proportion of variance explained and the specific analysis conducted.

As a proof of concept, we repeated a previously presented genome-wide association for VTE in participants of African ancestry (Heit et al., 2013). We compared the performance of the two approaches described above: (a) PCs derived from the “loadings” method and (b) PCs derived from the equivalent of the conventional method. Our results showed that using the “loadings” approach provided similar association results and controlled for inflation while controlling for fewer covariates and consequently fewer degrees of freedom. This method will need further validation using simulated data, but does seem promising nonetheless.

We have demonstrated that analysis of data across sites in research networks can expose subtle biases and stratification effects. The conventional approach of adjusting for the first number of PCs does not adequately adjust for the bias of platform and site. We recognize that in comparison to most meta analyses which use summary statistics for aggregation, we have both individual subject genotypes as well as information on genotyping platform and site. We hope our research study will serve as a reference for similar projects that attempt to control for confounders and ancestry in large genetic association studies.

## 5. Conclusion

In summary, we outline a general checklist for filtering genetic variants for conventional PCA to avoid the bias induced by platform and site as well as to avoid false-positive results due to the correlation between the PCs and the SNP genotypes. We have also proposed the “loadings” method as an alternative to the conventional method to derive PCs that control for bias due to the site and platform. Furthermore, we demonstrated the applicability of this new approach for the VTE genome-wide association analysis in genetic African ancestry eMERGE participants.

## Web resources

– eMERGE Coordinating Center genotyping data: http://emerge.mc.vanderbilt.edu/genotyping-data-released
– R package SNPRelate: https://github.com/zhengxwen/SNPRelate

## Funding

This study was supported by the following U01 grants from the National Human Genome Research Institute (NHGRI), a component of the National Institutes of Health (NIH), Bethesda, MD, USA: (1) U01HG006375 (Group Health/University of Washington); (2) U01HG006382 (Geisinger Health System); (3) U01HG006379 (Mayo Clinic); (4) U01HG006389 (Essentia Health, Marshfield Clinic Research Foundation, and Pennsylvania State University); (5) U01HG006388 (Northwestern University); (6) HG004438 (Center for Inherited Disease Research, Johns Hopkins University); (7) HG004424 (Broad Institute of Harvard and MIT); (8) U01HG006378, U01HG006385, U01HG006385 (Vanderbilt University and Pennsylvania State University); (9) U01HG006380 (The Mt. Sinai Hospital); (10) U01HG006828 (Cincinnati Children's Hospital Medical Center/Harvard); (11) U01HG006830 (Childrens Hospital of Philadelphia). Additional support was provided by a State of Washington Life Sciences Discovery Fund award to the Northwest Institute of Genetic Medicine (Gail P. Jarvik).

### Conflict of interest statement

The authors declare that the research was conducted in the absence of any commercial or financial relationships that could be construed as a potential conflict of interest.
