# Supplementary material for: Controlling for population structure and genotyping platform bias in the eMERGE multi-institutional biobank linked to electronic health records
Source: Front Genet. 2014 Nov 4;5:352. doi: 10.3389/fgene.2014.00352 (PMC4220165; doi:10.3389/fgene.2014.00352)
Supplement: Supplementary file 1 [file DataSheet1.PDF]

SUPPLEMENTARY FIGURES

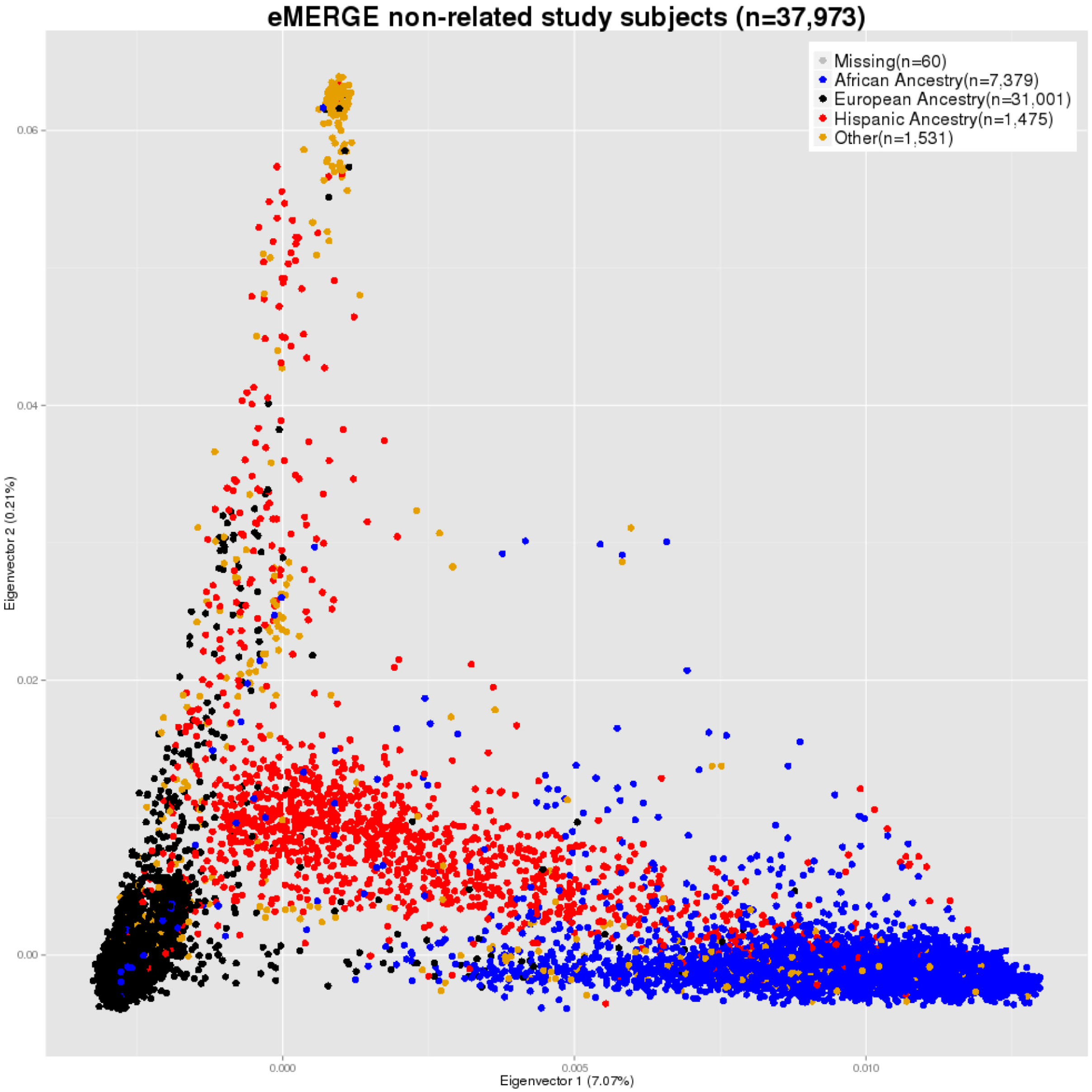

Figure S1. PC plots of PCs 1 and 2 for unrelated adults of eMERGE by self-reported race, using IMPUTE2 imputed data. Unrelated participants were not included unlike the prior PC analysis.
